# Assisting Visually Impaired People in the Public Transport System through RF-Communication and Embedded Systems †

**DOI:** 10.3390/s19061282

**Published:** 2019-03-14

**Authors:** Yessica Sáez, José Muñoz, Francisco Canto, Antony García, Héctor Montes

**Affiliations:** 1 Universidad Tecnológica de Panamá, El Dorado, Panama City 0819-07289, Panama; jose.munoz9@utp.ac.pa (J.M.); francisco.canto@utp.ac.pa (F.C.); antony.garcia@utp.ac.pa (A.G.); hector.montes1@utp.ac.pa (H.M.); 2 Centro de Estudios Multidisciplinarios en Ciencias, Ingeniería y Tecnología-AIP (CEMCIT-AIP), Panama City 0819, Panama; 3 Centre for Automation and Robotics CSIC-UPM, 28500 Madrid, Spain

**Keywords:** communication, embedded systems, mobility, MOVIDIS project, public transport, radiofrequency, visually impaired people

## Abstract

For a significant number of people with visual impairments, public transport plays an important role in productivity, community participation, and independence, since it may be the only feasible mobility option to participate in their education, work, medical care, food, and to attend many other places in their community. To use the public bus system safely, effectively, and autonomously, these people need to collect information about their physical environment and visible information at stops and terminals, such as timetables, routes, etc. Unfortunately, most people who are blind or visually impaired experience difficulties in getting on the right bus or getting off at the right destination. These situations usually force them to depend on other people that assist them in activities close to their homes, or settle for simpler jobs, or simply stay at home. Therefore, our efforts should aim to develop a system where technology is used to empower people with visual disabilities, allowing them to navigate autonomously in the public transport system. This paper presents a system based on radio frequency (RF) communication proposed within the framework of the MOVIDIS (Mobility for Visually Disabled People) research project (funded by the National Secretariat of Science, Technology and Innovation-SENACYT, under Grants No. 109-2015-4-FID14-073 and No. 99-2018-4-FID17-031), which provides an alternative to assist people with visual disabilities with their mobility in the public transport system. The various modules of this system communicate with each other by means of radio frequency and allow users to interact with buses and their respective stops. The first experimental results show that RF communication represents a viable option to help people with visual disabilities in public transport services.

## 1. Introduction

Disability is a natural and universal part of the human experience, sometimes permanent sometimes transitory. Every human being can experience, at some point in his/her life, some degree of disability, either due to a change in health or in the environment. According to the World Health Organization (WHO), 15% of the world’s population are estimated to live with some form of disability, and disability prevalence is on the rise [1].

In 2017, WHO’s statistics showed that 253 million people are visually impaired worldwide: 36 million are blind and 217 million have poor vision [2]. These individuals face physiological, psychological, social, and economic challenges that affect their quality of life and deprive them from performing many activities of daily living independently, being mobilization one of the most crucial.

For a significant number of visually impaired and blind persons, public transport plays an important role in productivity, community involvement, and independence, since it may be the sole viable mobility option to seek education, work, medical care, food and many other venues in their community. For the specific case of public bus system, individuals with visual disabilities need to gather information about their physical surroundings and the visible information that appears at bus stops and terminals such as schedules, routes, etc., in order to use public bus system safely, effectively, and autonomously. Unfortunately, most visually impaired and blind people may experience difficulties taking the correct bus and getting off at the right destination. These situations may force them to depend on others to drive them around, to stay at home, or to settle for simpler jobs and activities where they do not need to travel far. Hence, efforts should aim to develop a public transport system that people with visual disability can navigate autonomously, especially in developing countries, where the public transport services are very challenging.

Technological advancements could be used to empower people with visual disabilities. In this paper, an RF communication-based system proposed to help with the mobility of visually impaired and blind people to interact with the public transport system is described. The first experimental results show that RF communication represents a viable option for assisting people with a visual disability in transportation services.

Governments and non-governmental organizations have shown interest in investigating difficulties encountered by visually impaired and blind people when using public transport media. Several studies and reports that help with improving public transportation and making it usable for people with disabilities have been carried out. An example of such reports can be found in [3], where the main goal of this qualitative study was to identify mobility barriers faced by visually and hearing impaired people and to propose solutions and measures to help improve access to public transport for these two specific groups.

Many advanced public transportation services combining special handheld devices have been designed to provide the electronic orientation and dynamic information for visually impaired people. Examples include TYFLOSET (Tyflo Technologies Set) in the Czech Republic [4], NOPPA (Navigation and Guidance System for the Blind) in Finland [5], and PAVIP (Personal Assistant for Visual Impaired People) in Switzerland [6]. Mobile phones with specialized software installed have been suggested to be used as a medium to send sound messages to blind people, in combination with GPS (Global Positioning System), GSM (Global System for Mobile Communications), and Bluetooth technologies for location and communication purposes [7]. Furthermore, smartphone apps, such as Georgiephone [8], have been developed to provide the visually impaired with local bus information through a voice interface.

Other study proposed to install fixed base stations on the bus or tramway stops equipped with WiFi Access Point to communicate with the impaired people through a hand-held Personal Digital Assistant (PDA) with integrated voice synthesis [9]. In this work, the position of the bus is proposed to be monitored using different positioning systems, such as GPS.

While GPS technology has been tried in the past to make public transport navigation easier for the visually impaired people, this technology has not been very effective because of its lack of accuracy and slow response time up to nowadays. Early research has shown promising results for Radio Frequency (RF) communication as a replacement or supplement to GPS technology (depending of the action zones). A review is reported in [10] to evaluate the use of RF communication for the detection of objects and vehicles in motion, aiming to develop a system oriented to guide people with visual disabilities in the public transportation system. In [11], the authors proposed a system composed of two modules, one located in the bus and the other carried by the user. When a bus approaches a stop, the user must use its module to transmit an RF signal to the bus, which then will send back routes information to the user. Users then select their desired bus and the system uses an audio interface for guiding the user to the selected bus. Another study suggests the use of RF transceivers and microcontrollers in buses, and RF transceivers in a user’s module to detect and process the bus information [12]. In this work, users need to enter the desired bus and destination on his/her smartphone with the help of Google talkback app. This system also notifies the user about a bus arrival through the audio output.

The main disadvantage associated with the aforementioned work is that the system relies on the auditory cues for alerting the visually disabled person about a bus arrival and for guiding them to board the bus. This may be an issue in noisy environments since voice information could be difficult to decipher when many buses simultaneously arrive at the same station or bus stop. Another important aspect to take into account is that voice interfaces may interfere with people’s ability to hear important environmental sounds. In addition, the sounds emitted by the audio units can attract unwanted attention from people close to the user of the system. Therefore, one of the purposes of our work is to design a system that does not have audible information that can not be heard in noisy environments such as most bus stops that exist in most cities.

Many papers have been published and much work has been done on this issue, considering the intrinsic characteristics of each region or community. However, there is still a lot to be done. Therefore, this paper presents the results of a new and simple approach towards the conceptual design presented in [13,14], in which a transport system that uses RF communication between visually impaired people, bus stops, and buses, is proposed to improve the success rate of visually impaired to board correct buses and getting to the right destination. One of the main purposes of using RF is to take advantage of its short distance communication, since the communication between a bus and a bus stop will be possible only when the bus approaches the stop. During the journey of the person with visual disability, a GPS-based application could inform the person about his/her location, but when approaching the stop the GPS could fail, and that is where the short range communication of the RF technology plays a key role.

## 2. System Overview

**Figure 1:**
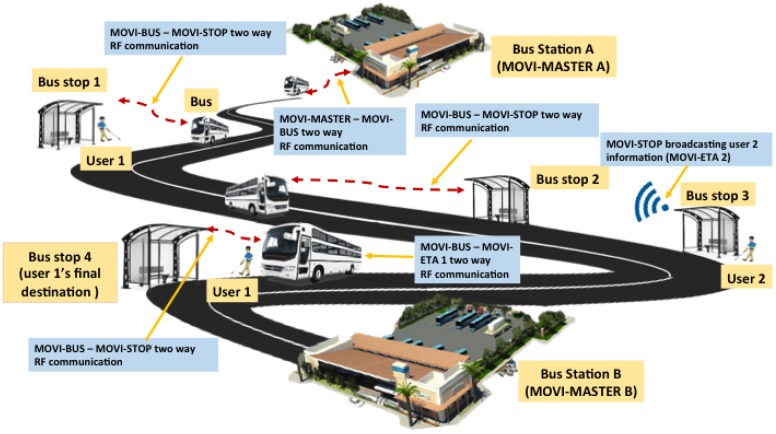
MOVIDIS conceptual working principle.

The proposed system has been called MOVIDIS (mobility for the visually disabled people). It is a dual system and the focus of interest on this paper consists of four RF modules: the bus module for the bus drivers (MOVI-BUS); the user module for the visually impaired persons (MOVI-ETA); the stop module (MOVI-STOP) located at the bus stops, which works as a communication link between the MOVI-ETA and the MOVI-BUS; and a module installed at the bus stations called the MOVI-MASTER. Figure 1 shows the conceptual working principle of the RF MOVIDIS system [10,13,14].

The MOVI-MASTER module is installed in every bus station where the RF MOVIDIS system works. This module is in charge of indicating the MOVI-BUS, which is the signal information (that is, first stop, direction, and final destination) that it must transmit for its interaction with the MOVI-STOPs located in each and every one of the stops distributed on its bus route. This information is stored in the MOVI-BUS module.

Consider the following: A bus carrying a MOVI-BUS module leaves Station A to Station B, using a predetermined route. This route has specific stops, which have been programmed on the MOVI-BUS module. As the bus goes through the route, it establishes communication with all the MOVI-STOPS at each of the stops on that route, sharing the bus ID and route details. When a person with visual disability carrying a MOVI-ETA module arrives at a bus stop with an installed MOVI-STOP module, he/she will let the MOVI-STOP know, through the MOVI-ETA module, what his/her final destination is. The MOVI-STOP is constantly communicating the user destination request, so any bus registered in the RF MOVIDIS system can learn that there is a MOVIDIS user at a specific stop. The MOVI-BUS checks if the destination matches one of its stops. If it does, the MOVI-BUS sends a warning signal to the driver to let him know that there is a user waiting for the bus at the stop. In addition, the MOVI-STOP must compare the destination selected by the user with the bus route and, if there is a match between the user’s destination and one of the stops of the bus route, it sends a warning signal to the user, letting him/her know that his/her bus is approaching and that he/she must prepare for boarding the next bus arriving at the stop. Once the user has boarded the bus, the MOVI-ETA will wait for a warning signal to let him/her know that he/she is approaching his final destination.

For the designs of the first modules, with special emphasis on the MOVI-ETA, the information collected from people with visual impairment and from institutions designed to provide aid to these people in Panama, such as SENADIS (National Secretariat for Disability) and IPHE (Panamanian Institute of Special Habilitation), was taken into account.

## 3. Materials and Methods

### 3.1. Participants

There were two types of participants in this study: experimenters and a person with a visual impairment. The experimenters were usually people between 20 and 22 years old, with heights ranging from 1.65 m to 1.85 m. The person with a visual disability was a 68 years old, male, approximately 1.79 m height. This person is blind due to Retinitis Pigmentosa [15], which refers to a group of inherited retinal diseases.

### 3.2. System Development

Since the MOVI-MASTER is the more complex module, this work considers the interaction among the MOVI-ETA, MOVI-STOP, and MOVI-BUS only. Therefore, we proceed to describe only these three modules in detail. Furthermore, even though in real environments the interaction among visually impaired people, bus drivers, and bus stops is a many-to-many relationship rather than a one-to-one, we considered, for technological simplicity, this system uses a one-to-one interactive RF communication system, in order to demonstrate the good functioning of the proposed system. In addition, we considered only one bus route with a limited number of stops between the initial and the final stop, but this can be also expanded to many more routes.

It is important to mention again that the devices used in all MOVIDIS modules are based on RF modules that operate within the so-called ISM (Industrial, Scientific, Medical) bands [16], such as 2.4 GHz and 433 MHz, which are free and do not require licensing. Therefore, since it is been proven that the 433 MHz frequency band has promising propagation characteristics for one-to-one communications using low-power wireless technologies [17], the signals to establish the communication between the different MOVIDIS system modules communicated through this frequency band. A TI- CC1101 transceiver (a device comprising both a transmitter and a receiver) was the chosen RF module due to its low energy consumption characteristics, the ability to use the received signal strength indicator (RSSI) and for being less prone to electromagnetic interference [18,19].

#### 3.2.1. MOVI-ETA

**Figure 2:**
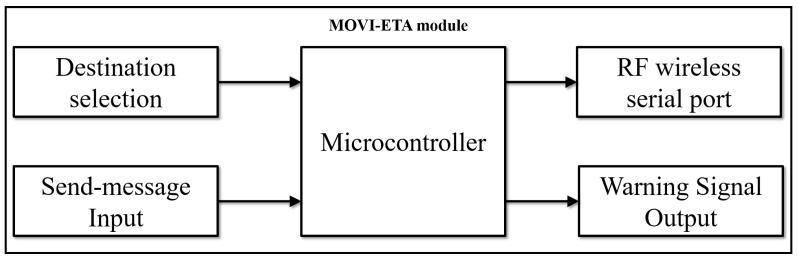
MOVI-ETA block diagram.

The MOVI-ETA module is based on an ATmega328P microcontroller (8 bits, 16 MHz, AVR architecture) and an HC-12 wireless serial port communication device that works in a similar way to a Bluetooth module. Its main function is to transmit the user’s data to the MOVI-STOP and at the same time to provide a suitable platform for people with visual disabilities so that they can select their destination and get a warning signal in the case of the arrival of a bus or the arrival to a requested stop. Figure 2 shows the block diagram of the MOVI-ETA module.

Each user is identified by a user ID consisting of 8 binary digits (1 byte). Thus, for simplicity, we assumed the system can manage 256 users, but it can be expanded to many more users in a future. This user ID has to be pre-programmed in each MOVI-ETA. The user selects his/her destination through switches. The number of possible stops depends on the number of switches, where each switch represents a binary number (1 or 0). For example, for three switches (as illustrated in Figure 2), the number of possible combinations (stops) is 8, that is, 000, 001, 010, 011, 100, 101, 110, and 111. Once the stop is selected through the switches, the user should press the push button to communicate his/her selection to the MOVI-STOP.

Through the HC-12, the MOVI-ETA sends two 8-bit codes (two bytes) to the MOVI-STOP: one byte to indicate the user ID and the other to specify the selected destination. Table 1 shows the information sent by the MOVI-ETA to the HC-12 located at the MOVI-STOP.

The user must press the push button to send the information to the MOVI-STOP through the HC-12, and he/she will receive a slight buzzer sound that will indicate him/her that the information was received by the MOVI-STOP. When a requested bus approaches the stop, the MOVI-STOP will let the user know of this event, by sending a signal that will activate the buzzer embedded in his/her MOVI-ETA.

#### 3.2.2. MOVI-STOP

The MOVI-STOP module is composed of two microcontrollers, two TI-CC1101 RF transceivers and an HC-12 device. This module is responsible for receiving the MOVI-ETA data and broadcasting them so that every MOVI-BUS passing close by the stop can receive it. It also works as a communication link between the MOVI-BUS and MOVI-ETA.

**Figure 3:**
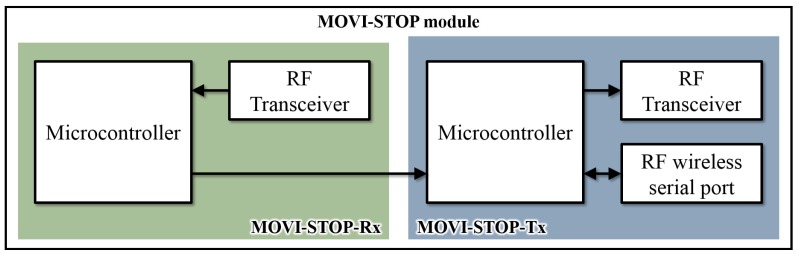
MOVI-STOP block diagram.

Since the TI-CC1101 only allows to transmit or receive but does not allow to do both simultaneously, two of these devices are used, one for transmission (MOVI-STOP-Tx) and one for reception (MOVI-STOP-Rx). In Figure 3 the MOVI-STOP module block diagram is shown.

The microcontroller of the MOVI-STOP-Tx module is connected to an HC-12, which will communicate with the HC-12 embedded at the MOVI-ETA. Once the user ID and final destination data are processed at the MOVI-STOP-Tx, this information is broadcasted through its TI-CC1101 and can be received by the TI-CC1101 connected to the microcontroller of the MOVI-STOP-Rx module. In this way, the MOVI-STOP-Rx will know about the user’s final destination.

The microcontroller of the MOVI-STOP-Rx module uses a proposed decision protocol (described below) based on the user’s information and the 7-byte data table described in Table 2, received through the TI-CC1101 from the MOVI-BUS and/or the MOVI-STOP-Tx.

All the information received from MOVI-STOP-Tx (user’s information) is stored in bytes 0, 1 and 2, while all information received from a MOVI-BUS is stored in bytes 3–6. MOVI-STOP-Rx receives all signals and identifies which one belongs to a specific MOVI-BUS. If the received data packet contains data in bytes 3, 4, 5 and 6, the MOVI-STOP will know that the data are coming from a MOVI-BUS; otherwise, it may be a signal from a nearby MOVI-STOP-Tx or its own MOVI-STOP-Tx. The MOVI-STOP-Rx compares the destination selected by the user with the one received from the bus and if it lies in between the initial stop and final stop values, it sends a signal to the MOVI-STOP-Tx so that it can inform the MOVI-ETA about the approaching bus via the HC-12.

It is important to mention that, since the MOVI-STOP-Tx broadcasts bytes 0, 1, and 2 through the TI-CC1101, this information can also be received by a MOVI-BUS that enters its coverage area. In addition, when a MOVI-ETA user is on the bus arriving at a stop, his/her MOVI-ETA will communicate with this stop, once within its coverage area. Thus, the user’s final destination will be sent to the MOVI-STOP-Tx. Therefore, if that stop happens to be the user’s final destination, the MOVI-STOP will send a signal to both the MOVI-BUS and the MOVI-ETA, and the buzzer will activate to alert the driver and the user that they are approaching the requested stop.

#### 3.2.3. MOVI-BUS

**Figure 4:**
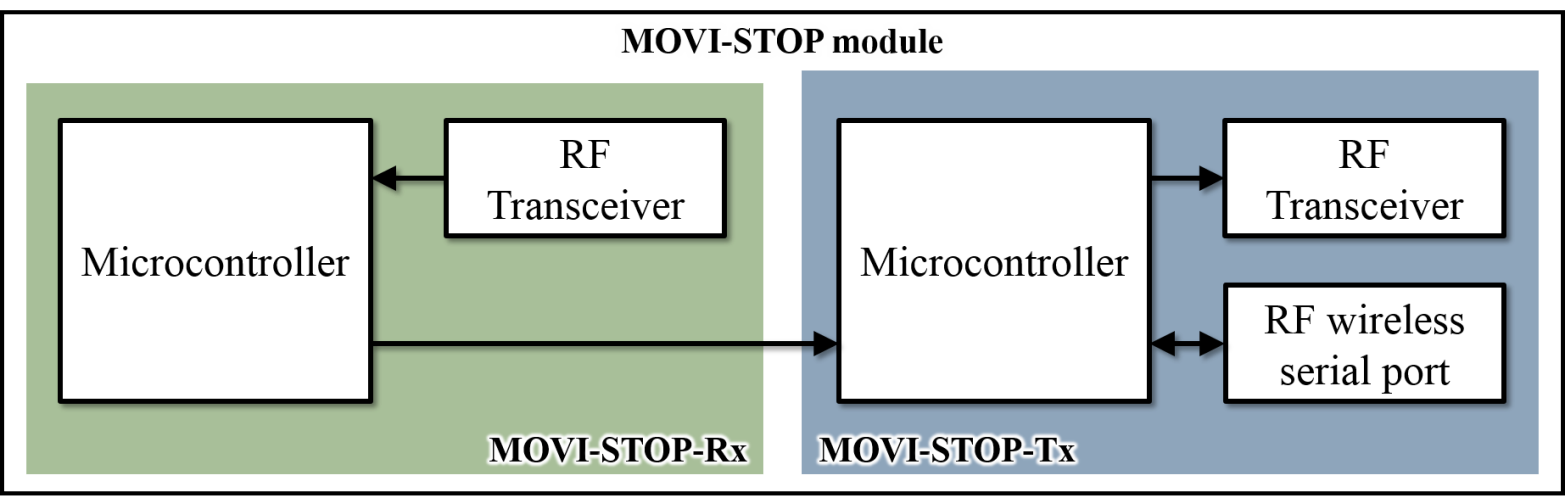
MOVI-BUS block diagram.

This module is based on two microcontroller and two TI-CC1101 RF transceivers. It is responsible for receiving the MOVI-STOP and MOVI-ETA data (via the MOVI-STOP). The MOVI-BUS module block diagram is shown in Figure 4.

It also has two sub-modules, one for transmission (MOVI-BUS-Tx) and one for reception (MOVI-BUS-Rx), and uses a 7-byte table similar to Table 2 to make decisions. It also has a buzzer to indicate the bus driver the presence of a MOVI-ETA user at a given stop. The MOVI-BUS is programmed so that it has stop table stored in it, which was already provided by the MOVI-MASTER.

The MOVI-BUS-Rx received a data table (similar to Table 2) from the MOVI-STOP (in this case, bytes 0, 1, 2 must be active). It compares the destination with its stops table and, if there is a match, it sends a buzzer signal to the driver so he can stop for the user waiting at the bus stop.

### 3.3. Testing Procedure

The validation of the proposed system was performed in four different phases. During the first phase, all the acquired equipment was tested to verify its correct operation and to have a point of reference for the operation of each device. Thus, a report on the technical aspects of every device was documented.

In the second phase, the communication between the MOVI-BUS and the MOVI-STOP modules was tested using specific stops (stationary tests), taking into account important RF communication aspects. These tests were conducted within a 224 m long street located at the *Universidad Tecnológica de Panamá* (UTP-Technological University of Panama) premises, where the MOVI-STOP and MOVI-BUS modules were mounted on a device that simulated the height at which they would be placed at the stop and the bus, respectively, in the line-of-sight range. In this phase, both modules were stationary aiming to certify the connectivity between them, and the operation of the system was examined considering several injections of data traffic.

During a third testing phase, we proceeded to conduct field tests for the communication link between the MOVI-BUS and the MOVI-STOP modules, where different environmental scenarios and vehicular mobility at different speeds were contemplated. This communication scenario was chosen to be tested separately because it has the particularity that one of the transceivers is in motion (MOVI-BUS). This is of great interest because validating the communication range of this module allows an early, adequate, and reliable vehicle detection (by the MOVI-STOP module) so that the bus can stop safely and comfortably upon a user’s request. Moreover, by comparing these results with those obtained in the stationary tests, it can be proven that the link is valid and useful for the MOVIDIS project.

The first three phases were conducted by experimenters only. Finally, the whole system was validated with the participation of a visually disabled (blind) person. In this case, the test scenario was the communication between MOVI-ETA module and MOVI-BUS module, using as a link channel the MOVI-STOP module between them. Thus, the user requested a bus (using MOVI-ETA), and then the MOVI-STOP notified the MOVI-BUS of the presence of the user at the stop. Then, the MOVI-STOP advised the user of the arriving bus (since the communication link between MOVI-STOP and MOVI-BUS was realized suitably). This test was performed at the UTP, Azuero Campus. The speed test was carried out 20 km/h, approximately.

## 4. Results

### 4.1. Stationary Tests

These tests were focused on establishing communication between the MOVI-BUS and the MOVI-STOP. The communication between the two modules was unidirectional, where the MOVI-STOP acted as a transmitter and the MOVI-BUS as a receiver. The transmission tests were carried out at discrete distances (nine measuring points, starting from 16 m until the maximum coverage range) along the selected section, taking 10 transmission measurements at each point. The maximum coverage range selected was 224 m. The tests were carried out successfully.

It is important to mention that the validations of the aforementioned parameters were verified at different times and environmental conditions in order to know the behavior of the signal throughout the day while considering different environmental conditions.

In this stage of testing, it was of interest to validate the following parameters:

- Coverage, that is, the geographical area in which the MOVI-BUS and MOVI-STOP can successfully communicate.
- The signal strength between the bus module (MOVI-BUS) and stop module (MOVI-STOP). To verify this intensity, the TI-CC1101 module located at the MOVI-STOP was used. This module has the built-in ability to estimate a received signal strength indicator (RSSI) [18,19]. The RSSI value was read continuously from the RSSI status register using the Texas Instrument design note 505 shown in [18].
- RF communication success rate, i.e., if the MOVI-STOP and MOVI-BUS are able to communicate during the entire duration of the test.

Table 3 summarizes the results.

It is clear that, in a wireless communication system there are many interferences, and if the systems are low cost, we will have even more interference. However, we performed experiments at different speeds to observe the quality of the signal during each experimental session. The idea was to know to what extent the proposed system could work properly. The RSSI measured had a range of −74 dBm to −80 dBm, approximately, with the antenna inside the vehicle, and from −68 dBm to −75 dBm, with the antenna on the outside of the vehicle, which represents a good range of coverage [19]. Additionally, to avoid possible interferences, these first experiments were performed at distances between 16 m and 224 m, being sufficient to validate the proposed system. In future work, we will expand the coverage area, and for this we will adapt amplifiers in the modules at bus stops and buses.

### 4.2. MOVI-BUS–MOVI STOP Field Tests

The place selected for the field tests was the old 1 km-long runway at the Pedasí airport, located in the province of Los Santos, Panamá. The main reasons for choosing this place were: easy access, no interference with other wireless devices, no presence of natural users that exist in the normal roads in the cities and towns, and flexible hours of use, which allowed testing different speeds at any time.

These tests were focused on establishing communication between the MOVI-BUS and the MOVI-STOP. The communication between the two modules was bidirectional, where both the MOVI-STOP and MOVI-BUS acted as a transmitter and receiver at the same time. However, only the data received by the MOVI-STOP were recorded for statistical purposes.

These tests were performed considering four different speeds: 40, 60, 80 and 100 km/h, the last being the maximum allowed by the *Autoridad de Tránsito y Transporte Terrestre* (ATTT—Transit and Land Transport Authority of Panama). Initially, multiple experiments on different days were performed, aiming to validate the functioning of the modules designed and developed in the project while considering different weather/climatology conditions. Finally, a series of five experiments for each speed were carried out to validate the results previously obtained. The final results are similar to those obtained in the previous tests.

To simulate the bus, an experimenter’s personal car was used and the MOVI-BUS was located both inside and outside the car, to simulate two different positions of its antenna. In this case, the battery of the vehicle was used as a power source. It is important to mention that the vehicle used for testing had cruise control, which helped to automatically control its speed.

**Figure 5:**
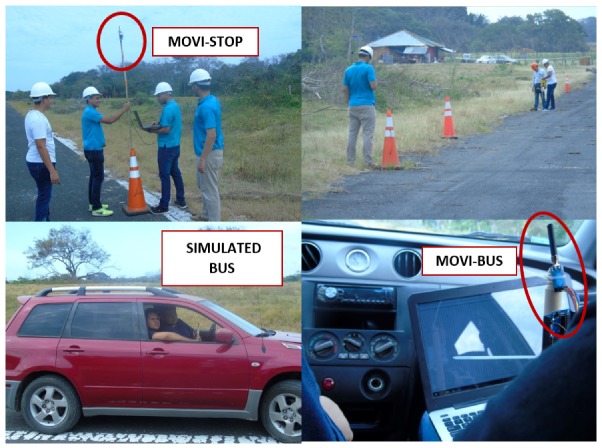
MOVI-BUS–MOVI-STOP field tests.

The MOVI-STOP was again mounted on a device simulating the bus stop (see Figure 5). Since previous results from stationary tests indicated that the MOVI-STOP and MOVI-BUS modules started communicating when the bus was 224 m apart, we used this distance as a reference to start transmitting data. However, since the new scenario involved mobility of the MOVI-BUS, it was noted that, at 100 km/h, these modules started communicating at 81 m. A laptop was used as a power source for the MOVI-STOP module.

During these tests, it was of interest to validate the following parameters:

- The average signal strength between the bus module (MOVI-BUS) and stop module (MOVI-STOP). Again, the TI-CC1101 module located at the MOVI-STOP was used to estimate the RSSI.
- The average amount of successful transmissions. For each speed, the MOVI-BUS was programmed to transmit information every 100 ms.
- The average amount of bytes with errors. For each speed, the transmission consisted of 60 packets of 1 byte each.
- Location of the MOVI-BUS antenna (inside or outside the vehicle).
- RF communication success rate, that is, if the MOVI-STOP and MOVI-BUS are able to communicate during the entire duration of the test.

Table 4 summarizes the results of the data stored in the MOVI-STOP module during the field tests.

### 4.3. System Demonstration

The MOVI-ETA, MOVI-STOP, and MOVI-BUS modules were built within the framework of MOVIDIS project, as mentioned above. The communication scenario between the MOVI-ETA and the MOVI-BUS modules, using as a link channel the MOVI-STOP module between them, was tested. In this case, the main participant was the person with a visual disability described in the previous section. The specific scenario where the user requested a bus using the MOVI-ETA was several times. In this case, the MOVI-ETA was able to successfully inform the MOVI-STOP about the user’s request and the MOVI-STOP successfully communicated the MOVI-BUS that a MOVI-ETA user was requesting its bus service. Then, the MOVI-STOP was also able to communicate with the MOVI-ETA, to alert the user of the bus arrival.

**Figure 6:**
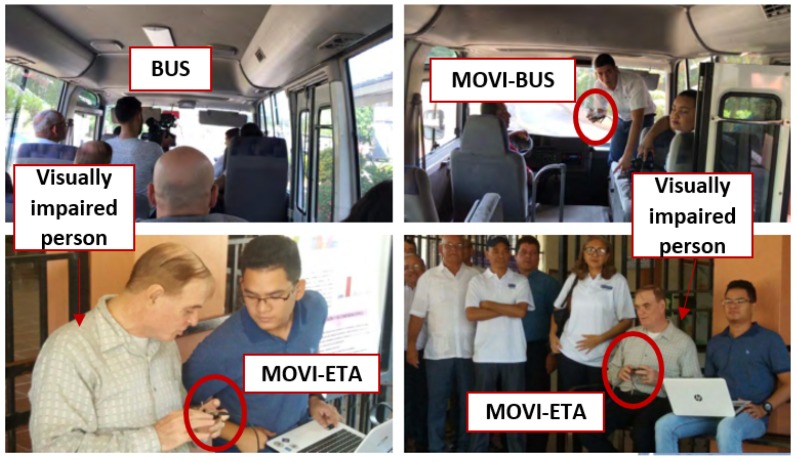
MOVIDIS system demonstration.

It is important to mention that the effective communication distance was certainly less than in the field tests, because of the electromagnetic interference at the demonstration place. Besides that, we had to guarantee the security of the visually impaired person and special invitees. Thus, for this specific test, the only objective was to validate the communication between the three modules, thus no errors, RSSI, and amount of data transmissions were taken into account. In addition, for safety purposes, the MOVI-BUS module’s antenna was located inside the bus, which was traveling at approximately 20 km/h. Figure 6 illustrates some photos during the system demonstration.

## 5. Discussion and Future Scope

The RF-based communication MOVIDIS system is a low-cost and simple solution to assist visually impaired people in their autonomous mobility when using the public transport system. Using the interactive RF-based communication modules, visually impaired people can request a bus service by giving information to bus drivers, boarding the correct bus, and reaching their destination easily.

The interactive RF-based communication was successful in all tests, meaning the microcontrollers, RF transceivers, and other electronic devices are suitable for the proposed system. However, we confirm that the wireless communication is substantially influenced by the mobility of the bus. This is proven by the fact that, during the stationary tests, the bus and stop modules could establish communication when they were 224 m apart. Nonetheless, once mobility of the bus was considered, at a high speed (100 km/h), the two aforementioned modules established communication when they were 81 m apart. However, this could be improved by acquiring more powerful equipment and, therefore, more expensive than those acquired in the project. Thus, recommendations can be made in order to increase the transmission range between modules, among other technical details of interest.

The location of the antenna of the MOVI-BUS module was proven to influence the performance of the system. During the field tests, it could be noticed that, by locating the antenna outside the “bus”, there was better signal intensity measured at the stop, as well as more successful message transmissions and less amount of bytes with errors.

In the field test, considering the last experimental series, we observed that the average percentage number of bytes with errors is highest at 60 km/h, which disagrees with the previous experiment series. This is an unexpected result, which needs further studies and more control during experimentations. However, notice that the RSSI at this speed is smaller than for the rest of the tests. Thus, we must emphasize that some small errors, related to the position and orientation of both the bus module and the stop module, might have occurred during the last experimentations. However, the average RSSI is within the coverage range for an acceptable data rate for this wireless communication system [18].

During the system demonstration tests, the wireless communication distance for the link between the MOVI-STOP and MOVI-BUS modules was less than in the field tests. This result might be attributed to the bus windows being closed, the MOVI-BUS being located inside the bus, and the university road being surrounded by large buildings. Although the wireless communication distance was shorter, the participant (the person with a visual disability) was able to successfully receive a notification of the arriving bus. We observed the bus traveling at a speed of approximately 20 km/h when approaching the stop, which was able to stop the bus quickly and easily, after being notified of the presence of the MOVI-ETA user at the simulated stop.

Future work includes further improvements that need to be made to the system including improving the stability of the wireless communication devices to reduce errors and to increase the number of successful transmissions. In addition, the system has to be upgraded and tested to a multi-user system that meets the requirements of a larger group of visually impaired people taking multiple buses at a given time and multiple buses arriving at a particular bus stop. In this case, communication aspects such as interference and distortion must be studied. Additionally, the first tests of the new RF modules implemented in real controlled scenarios (bus routes) will be carried out to know the usability of the system with people with visual disabilities. It will be considered to design surveys for users with visual disabilities that cooperate with the project, considering the ethical aspects of the country. These surveys will be implemented during the training phase of the system, as well as after its validation on the bus route. For this, the Likert scale will be taken into account to design a survey to measure the attitude and to know the degree of conformity of people with visual disability in the use of the proposed system.

## 6. Conclusions

The main aim of the MOVIDIS project is helping visually disabled people to become more independent in their daily activities by developing a system to aid them when using the public transport system. Thus, this work presents a new approach to provide autonomous mobility in the public transport to visually impaired persons by means of RF communication and embedded systems. This new prototype has many advantages—cheap, user-friendly, modular, and no audio cues, among others—which make it a good alternative to the current approaches.

An interactive RF-based communication aid system for the visually impaired people to use the public transport was presented and validated in this study. Test results indicate that this system could help users to successfully request their desired buses, using the interactive communication modules, which worked most favorably when the buses were traveling at low speeds. The system concept and its modules are simple and easy to understand. The field tests and the system demonstration showed the feasibility of the proposed system and provided a reference basis for developing a more complex system for aiding visually impaired bus users in the future.

The main electronic devices used were ATmega328P microcontrollers, HC-12 series communication, and TI-CC1101 RF transceivers; however, for each electronic card designed for each module, we added electronic support and dedicated interconnections according to the electronic design of the system. In addition, dedicated algorithms were designed so that the RF communication system functions as previously established in the proposed methodology. The arrangement of the antennas was adequate during the multiple experiments carried out by the experimenters, with the aim of avoiding interferences as much as possible.

In addition, compared to other works, one of the main contributions of the solution presented in this article is the addition of the MOVI-STOP module. Because it is possible to have a fixed power supply at some bus stops, or at least to install battery banks recharged by solar panels, it is possible that the MOVI-STOP can have a high output power in its transmitter. The MOVI-BUS can also be powered by the vehicle’s battery, thus a high output power is also possible at its transmitter. This allows the minimum distance for establishing the communication link between the MOVI-BUS and the MOVI-STOP to be sufficiently large to allow the MOVI-ETA (which will have a power limitation) to receive notification (on time) of the bus approaching and, therefore, the user can react accordingly.

With the implementation of the proposed system, the life of the visually impaired people will change, since it gives them the opportunity to reach their destination and to contribute positively to their society by overcoming their obstacles related to the ability to move freely and without the help of a tutor. 

## Figures and Tables

**Table 1:** Information transmitted/received by the MOVI-ETA module.

TRANSMITTED (HC-12)			RECEIVED (HC-12)			
	User ID	Destination	Letter →	R	A	X
Size→	1 Byte	1 Byte	Function→	Message	Bus arrival	Turn off
	(8 bits)	(8 bits)		received		the buzzer

**Table 2:** MOVI-STOP-Rx data table received from MOVI-BUS and/or MOVI-STOP-Tx.

Byte	0	1	2	3	4	5	6
Name	Stop ID	User	Destination	Bus ID	Route	Initial Stop	Final Stop
Range	0–255	0–255	0–255	0–255	0–255	0–255	0–255

**Table 3:** Stationary test results.

**Time of test**	Morning	Afternoon	Night
**Participant**	Experimenters	Experimenters	Experimenters
**Weather**	Cloudy	Sunny	Cloudy
**Maximum communication range: 224 m**			
**Avg. RSSI (dB)**	−86	−87	−89
**Avg. RF Communication success rate (%)**	100	100	100
**Minimum communication range tested: 16 m**			
**Avg. RSSI (dB)**	−74	−74	−74
**Avg. RF Communication success rate (%)**	100	100	100

**Table 4:** MOVI-BUS–MOVI STOP field test results.

**Speed**	40 km/h	60 km/h	80 km/h	100 km/h
**Vehicle**	Mitsubishi Outlander 2003	Mitsubishi Outlander 2003	Mitsubishi Outlander 2003	Mitsubishi Outlander 2003
**Weather**	Sunny	Sunny	Sunny	Sunny
**Participants**	Experimenters 1, 2, 3	Experimenters 1, 2, 3	Experimenters 1, 2, 3	Experimenters 1, 2, 3
	**Antenna in**			
**Avg. RSSI (dBm)**	−74.78	−79.76	−75.09	−79.78
**Avg. successful transmissions**	25.5	14.2	12	8.25
**Avg. percentage of bytes with errors (%)**	3.67	15.43	0.7	5.82
**RF communication success rate (%)**	100	100	100	100
	**Antenna out**			
**Avg. RSSI (dBm)**	−68.37	−74.79	−71.23	−71.44
**Avg. successful transmissions**	28	21	17	13.75
**Avg. percentage of bytes with errors (%)**	0	1.84	0	0.06
**RF communication success rate (%)**	100	100	100	100
